# S08 Sharing experiences in local PA policy development and monitoring, to increase collaboration, efficiency, and inclusiveness

**DOI:** 10.1093/eurpub/ckae114.231

**Published:** 2024-09-26

**Authors:** Peter Gelius

**Affiliations:** Institut des Sciences du Sport, Université de Lausanne

## Abstract

The importance of local governments' engagement in physical activity (PA) promotion has been widely acknowledged and described at international and national levels, by governmental and non-governmental organizations.

Generally, public policies can positively influence PA determinants and local PA policies in particular can have a more direct and sustainable positive impact on the target populations.

However, the monitoring of the development and implementation of these (legally binding or voluntary based) local initiatives has so far been lagging behind the expected or needed coverage and / or consistency.

The current symposium aims to present how local PA policy assessment can contribute to enlightenments in regards to policy monitoring scaling-up, increasing cross-sectoral collaboration to increase PA policy effectiveness and PA services accessibility and highlighting the gap between goals and practices in local PA policy targeting diverse population subgroups.

The presentations will be followed-up by a Q&A session focusing on aspects related to the importance of local PA policy monitoring and evaluation and the actions local governments can uptake in order to shift from experimental to routine policy monitoring and how academia can support this transition.

